# Evaluating the impact of donor eGFR and HLA-DR mismatch on graft survival in living donor kidney transplants

**DOI:** 10.3389/fneph.2024.1518791

**Published:** 2025-01-07

**Authors:** Pooja Budhiraja, Jesse D. Schold, Rocio Lopez, Susana Arrigain, Bruce Kaplan

**Affiliations:** ^1^ Department of Medicine, Mayo Clinic Arizona, Phoenix, AZ, United States; ^2^ Department of Surgery, University of Colorado Anschutz Medical Campus, Aurora, CO, United States; ^3^ Department of Epidemiology, University of Colorado Anschutz Medical Campus, Aurora, CO, United States; ^4^ Department of Medicine, University of Colorado Anschutz Medical Campus, Aurora, CO, United States

**Keywords:** HLA-DR, HLA matching, living donor kidney transplant, eGFR, graft survival

## Abstract

**Background:**

This study assesses the impact of human leukocyte antigen (HLA)-DR mismatch and donor-estimated glomerular filtration rate (eGFR) on outcomes of living donor kidney transplantation (LDKT), which are especially relevant to the availability of multiple donors and paired kidney exchanges.

**Methods:**

Using data from the Scientific Registry of Transplant Recipients (SRTR), we retrospectively analyzed graft survival in adult LDKT recipients transplanted between January 2013 and September 2022. Recipients with 0 HLA-DR mismatches were compared to those with 1-2 HLA-DR mismatches. Cox models assessed the association between donor eGFR and graft and patient survival, stratifying by a) HLA-DR mismatches, and b) HLA-DR mismatches and recipient age.

**Results:**

Among 44,080 recipients, 7,195 had 0 HLA-DR mismatches and 36,885 had 1-2 HLA-DR mismatches. The recipients’ mean age was 49.1 for the 0 HLA-DR mismatch group and 50.4 for the 1-2 HLA-DR mismatch group. The donors’ mean age was 43.1 and 43.8, with an eGFR of 101.0 and 99.9 ml/min, respectively. A higher donor eGFR was associated with better graft survival. Stratified analyses showed higher donor eGFR levels reduced the risk of graft loss in cases with DR mismatch (p < 0.001) but not in cases without HLA-DR mismatch (p = 0.81). This effect was significant for recipients aged 18-39 and over 60. Similar results were observed for patient survival.

**Conclusions:**

Higher donor eGFR was associated with lower risks of graft loss and patient death in the HLA-DR mismatch group but not the 0 HLA-DR mismatch group. These results emphasize the importance of considering both HLA-DR matching and donor kidney function, particularly for younger recipients to avoid sensitization for future transplants.

## Introduction

Kidney transplantation remains the most effective treatment for end-stage renal disease, offering significant improvements in quality of life and survival compared to dialysis. However, the long-term success of a transplant is influenced by multiple factors related to both the donor and the recipient. Despite advancements in managing acute rejection, chronic allograft nephropathy remains a significant cause of late graft loss.

Living donor kidney transplantation (LDKT) is associated with improved patient and graft survival compared to deceased donor kidney transplantation (DDKT). Notably, the risk of graft failure in a zero mismatched DDKT is comparable to that of a living donor kidney transplant with five or six mismatches (1).

This highlights the potential of LDKT to achieve favorable outcomes even in less-than-ideal human leukocyte antigen (HLA) match scenarios, attributed to the enhanced condition and immediate availability of the donated organ. It underscores the controversial significance of waiting for HLA matching in LDKT, suggesting that other factors may also play critical roles in transplant success.

Research examining factors impacting LDKT survival has suggested that a donor’s renal function, donor age, and compatibility of HLAs significantly affect outcomes (2–4). While some studies affirm the association between HLA matches and graft outcomes in LDKT (4–6), others dispute the relevance of such matching when compared to other factors when selecting living donors (7, 8). Thereby suggesting that donor-estimated glomerular filtration rate (eGFR) and other donor factors may play a more critical role in the prognosis of kidney transplants, potentially mitigating the risks associated with HLA mismatches.

Class II HLA mismatches are considered more immunogenic than Class I mismatches, leading to a more robust and diverse immune response capable of driving both cellular and humoral rejection processes. The impact of HLA-DR mismatches is particularly notable, with some studies suggesting they are a significant risk factor for graft survival, with a much greater effect than HLA-B or -A. This highlights the greater effect of HLA-DR matching compared to HLA-B or -A (4, 9).

Donor-recipient matching has become increasingly important, particularly with the option of choosing between donors and the increasing popularity of paired kidney exchanges. While HLA-DR mismatches have been widely studied, there is limited understanding of their combined impact with donor kidney function on long-term graft outcomes. This study aims to assess the impact of HLA-DR mismatch in conjunction with donor eGFR on the outcomes of LDKT. Given the evolving landscape of kidney transplant outcomes and the pivotal role of renal function metrics, our research aims to dissect the complex relationship between donor eGFR, HLA mismatches, and recipient age.

## Methods

### Data description and study population

This study used data from the Scientific Registry of Transplant Recipients (SRTR). The SRTR data system includes data on all donors, wait-listed candidates, and transplant recipients in the US, submitted by the members of the Organ Procurement and Transplantation Network (OPTN). The Health Resources and Services Administration (HRSA), US Department of Health and Human Services, provides oversight of the activities of the OPTN and SRTR contractors. The data reported here have been supplied by the Hennepin Healthcare Research Institute (HHRI) as the contractor for the SRTR. The interpretation and reporting of these data are the responsibility of the author(s) and in no way should be seen as an official policy of or interpretation by the SRTR or the US Government. This retrospective cohort study used the standard analysis files from the SRTR as of September 2022.

**Figure 1:**
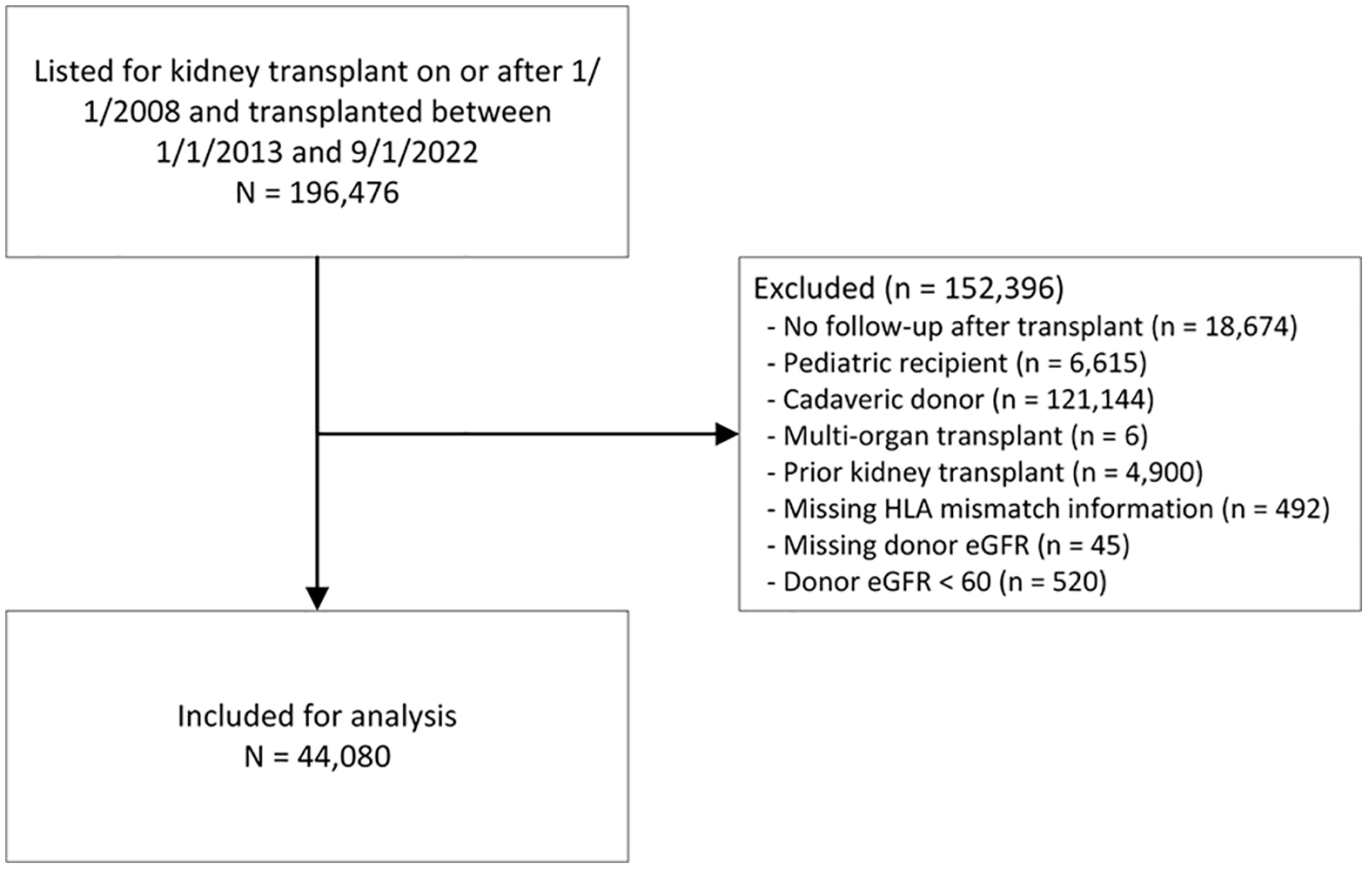
Patient selection flow chart. eGFR, estimated glomerular filtration rate; HLA, human leukocyte antigen.

We identified subjects listed for kidney transplantation on or after 1 January 2008, and who received a kidney transplant between 1 January 2013 and 1 September 2022. We included listings from 2008 onwards because this is the era when the results from calculated panel reactive antibody (cPRA) tests began to be populated in the registry. The following exclusions were made: no post-transplant follow-up, younger than 18 at the time of transplant, cadaveric kidney donor recipient, multi-organ transplant, prior kidney transplant, missing HLA mismatch information, missing donor eGFR, and donor eGFR less than 60 (**Figure 1**).

### Definitions and outcome measures

We calculated donor eGFR using the 2021 race-free Chronic Kidney Disease Epidemiology Collaboration (CKD-EPI) creatinine equation (10). We also calculated donor eGFR using the 2009 race-included (11) creatinine equations to assess whether the results held, as this was the formula used for the majority of the study period; these results are presented in **Supplementary Material 1**.

2021 CKD − EPI (race free) (10)


$$\begin{array}{c} \text{eGFRcr }=\;142\text{ x min }\left(\text{Scr}/\kappa ,\;1\right)\;\alpha \;x\;max\;\left(Scr/\kappa ,\;1\right)\\ -1.200\;x\;0.9938Age\;x\;1.012\;\left[if\;female\right] \end{array}$$

2009 CKD − EPI creatinine equation (11)


$$\begin{array}{c} eGFR\;=\;141\;\times \;min\left(Scr/\kappa ,\;1\right)\alpha \;\times \;max\left(Scr/\kappa ,\;1\right)-1.209\;\\ \times \;0.993Age\;\times \;1.018\;\left[if\;female\right]\;\times \;1.159\;\left[if\;black\right] \end{array}$$

where Scr is serum creatinine, κ is 0.7 for women and 0.9 for men, α is −0.329 for women and −0.411 for men, min is the minimum of Scr/κ or 1, and max is the maximum of Scr/κ or 1.

We also calculated the living kidney donor profile index (LDKPI) (12). For example, a donor with an LKDPI of 20 indicates that live donor transplantation using a kidney from this donor would have an expected risk equivalent to deceased donor transplantation from a donor with a KDPI of 20. The factors included in the equation are the donor’s age, eGFR, BMI (body mass index), African American ethnicity, history of cigarette use, systolic blood pressure (SBP), whether the donor and recipient are both male, ABO incompatibility, whether the donor and recipient are unrelated, the number of HLA-B mismatches, the number of HLA-DR mismatches, and the donor/recipient weight ratio (D/RWR).

Our primary outcomes were time to post-transplant patient and graft survival. We defined kidney graft survival time as the number of months from transplantation to irreversible graft failure, kidney re-transplantation, or patient death. We defined patient survival as months from transplantation until death or recipient censoring cohort date. The recipient censoring cohort date was used to censor mortality, and the minimum between the recipient censoring cohort date and the last graft follow-up date was used to censor kidney graft loss. All follow-up was truncated at 5 years. The cohort censoring date for our analysis cohort was September 2, 2022.

### Missing data

Data were missing for the following variables: recipient education (2.1%), peak cPRA (1.2%), LDKPI (0.54%), recipient BMI (0.41%), dialysis duration at transplant (0.38%), donor BMI (0.26%), on dialysis (0.18%), donor/recipient weight ratio (0.15%), donor diabetes (0.12%), donor hypertension (HTN) (0.08%), recipient’s primary insurance (0.05%), disease etiology (0.03%), and diabetes type (0.02%).

We used multivariate imputation by chained equations to impute five datasets with complete data. The multiple imputation model included the following characteristics: recipient sex, age category, race/ethnicity, BMI category, education, primary insurance, disease etiology, dialysis duration category, peak cPRA category, donor sex, donor age category, donor BMI category, donor blood type, and donor eGFR, ABO incompatibility, blood relationship between donor and recipient, donor/recipient weight ratio, and presence of HLA-DR mismatch.

### Statistical analysis

We summarized continuous variables using means and standard deviations, and categorical factors using frequencies and percentages. We compared recipients with and without HLA-DR mismatches using t-tests for continuous variables and Pearson’s chi-square tests or Fisher’s exact tests for categorical variables. Donor characteristics were also compared by HLA-DR mismatch.

Using multivariable Cox regression models, we evaluated the association of donor race-free eGFR and HLA-DR mismatch with time to graft loss and patient death. To consider the non-linear effects of eGFR, we used a restricted cubic spline term with three equally spaced knots. We tested interactions between donor eGFR and the presence of HLA-DR mismatch and built models stratified by HLA-DR mismatch. All the multivariable models were adjusted for recipient age category, race/ethnicity, BMI category, education, insurance, disease etiology, peak cPRA category, duration on dialysis category, donor/recipient weight ratio, donor and recipient both being male, donor and recipient having the same race/ethnicity, donor and recipient being related, and ABO blood group (ABO) incompatibility. All the Cox regression models were fitted to each of the five imputed datasets, and the parameter estimates were combined. To display the effect of eGFR on graft loss and patient death, we plotted adjusted hazard ratios at eGFR levels 80 to 120 mL/min/1.73m2 using eGFR equal to 90 as the reference level.

We tested the following interactions in the models: donor eGFR * HLA-DR mismatch, and donor eGFR * HLA-DR mismatch * recipient age group. In addition, we fitted stratified analyses to evaluate the association between eGFR and outcomes as follows: stratified by HLA-DR mismatch and stratified by HLA-DR mismatch and recipient age group.

Finally, we performed a sensitivity analysis by replicating the above using the 2009 race-included eGFR creatinine equation.

All tests were two-tailed and performed at a significance level of 0.05 using SAS 9.4 software (SAS Institute, Cary, NC). The Colorado Multiple Institutional Review Board approved the study and consent waiver was obtained. The data reported here have been supplied by the HHRI as the contractor for the SRTR.

## Results

### Characteristics of living donor kidney transplant recipients by DR mismatches

**Figure 2:**
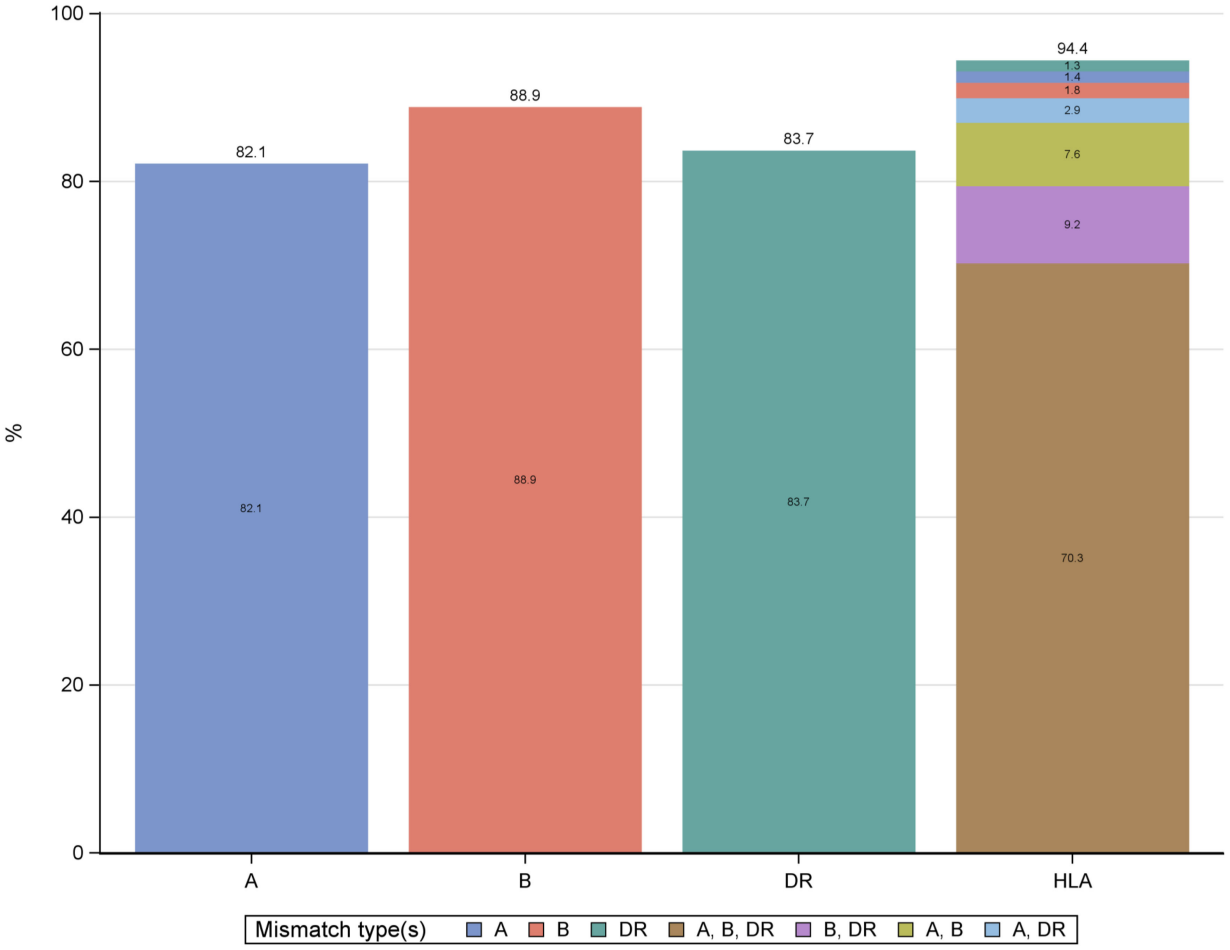
Frequency of HLA mismatches. HLA, human leukocyte antigen.

The study included 44,080 recipients of LDKT who were primarily analyzed for HLA-DR mismatches (**Figure 1** **).** The distribution of mismatches was 82% for HLA-A, 88.9% for HLA-B, and 83.7% for HLA-DR, with 70.3% of recipients showing mismatches across HLA-A, HLA-B, and HLA-DR (**Figure 2**). The analysis focused on the HLA-DR mismatches.

The LDKT recipients with 0 HLA-DR mismatches were slightly younger on average compared to those with 1-2 HLA-DR mismatches (mean age 49.1 vs 50.4 years; p < 0.001) (**Table 1**). In addition, the 0 HLA-DR mismatch group had a higher proportion of women than the 1-2 HLA-DR mismatch group (39.6% vs. 36.2; p < 0.001). The majority of recipients were non-Hispanic white, with 62.4% in the 0 HLA-DR mismatch group vs. 64.1% in the 1-2 HLA-DR mismatch group. The 0 HLA-DR mismatch group had a higher proportion of Hispanic white recipients (19.1%) and a lower proportion of Black recipients (10.2%) compared to the 1-2 HLA-DR mismatch group (15.1% Hispanic white and 13.4% Black) (p < 0.001). The mean BMI was lower in the 0 HLA-DR mismatch group (27.7 kg/m2) compared to the 1-2 HLA-DR mismatch group (28.1kg/m2) (p < 0.001).

**Table 1:** Characteristics of living donor kidney transplant recipients by HLA-DR mismatches.

Factor	Overall (N=44,080)		0 HLA-DR mismatches (N=7,195)		1-2 HLA-DR mismatches (N=36,885)		p-value
	N missing	Statistics	N missing	Statistics	N missing	Statistics	
Age at transplant (years)	0	50.2 ± 14.4	0	49.1 ± 14.7	0	50.4 ± 14.3	***<0.001^a2^***
Age at transplant (years)	0		0		0		***<0.001^c^***
18 - 39		10,986 (24.9)		2,035 (28.3)		8,951 (24.3)	
40 - 49		8,652 (19.6)		1,350 (18.8)		7,302 (19.8)	
50 - 59		11,100 (25.2)		1,743 (24.2)		9,357 (25.4)	
60+		13,342 (30.3)		2,067 (28.7)		11,275 (30.6)	
Sex	0		0		0		***<0.001^c^***
Female		16,217 (36.8)		2,849 (39.6)		13,368 (36.2)	
Male		27,863 (63.2)		4,346 (60.4)		23,517 (63.8)	
Race/Ethnicity	0		0		0		***<0.001^c^***
Non-Hispanic white		28,131 (63.8)		4,487 (62.4)		23,644 (64.1)	
Hispanic white		6,939 (15.7)		1,374 (19.1)		5,565 (15.1)	
Black		5,698 (12.9)		737 (10.2)		4,961 (13.4)	
Other		3,312 (7.5)		597 (8.3)		2,715 (7.4)	
Education*	906		129		777		***0.005^c^***
High school or less		14,346 (33.2)		2,465 (34.9)		11,881 (32.9)	
Some college		11,494 (26.6)		1,844 (26.1)		9,650 (26.7)	
College or more		17,334 (40.1)		2,757 (39.0)		14,577 (40.4)	
Primary insurance*	20		1		19		***<0.001^c^***
Private		24,501 (55.6)		4,054 (56.4)		20,447 (55.5)	
Medicare		16,807 (38.1)		2,620 (36.4)		14,187 (38.5)	
Medicaid/CHIP		1,919 (4.4)		363 (5.0)		1,556 (4.2)	
Other		833 (1.9)		157 (2.2)		676 (1.8)	
BMI (kg/m2) *	179	28.0 ± 5.5	23	27.7 ± 5.6	156	28.1 ± 5.4	***<0.001^a2^***
BMI (kg/m2) *	179		23		156		***<0.001^c^***
<20		2,494 (5.7)		493 (6.9)		2,001 (5.4)	
20 - 24.9		11,262 (25.7)		1,989 (27.7)		9,273 (25.2)	
25 - 29.9		14,851 (33.8)		2,300 (32.1)		12,551 (34.2)	
30 - 34.9		10,304 (23.5)		1,628 (22.7)		8,676 (23.6)	
35+		4,990 (11.4)		762 (10.6)		4,228 (11.5)	
Disease etiology*	12		1		11		***<0.001^c^***
Glomerulonephritis		12,686 (28.8)		2,230 (31.0)		10,456 (28.4)	
Diabetes		11,092 (25.2)		1,720 (23.9)		9,372 (25.4)	
Polycystic Kidney Disease		5,911 (13.4)		751 (10.4)		5,160 (14.0)	
Hypertension		7,113 (16.1)		1,164 (16.2)		5,949 (16.1)	
Other		7,266 (16.5)		1,329 (18.5)		5,937 (16.1)	
Diabetes type*	10		3		7		***<0.001^c^***
No diabetes		30,787 (69.9)		5,145 (71.5)		25,642 (69.5)	
Type I		1,971 (4.5)		349 (4.9)		1,622 (4.4)	
Type II		11,002 (25.0)		1,651 (23.0)		9,351 (25.4)	
Type other		213 (0.48)		30 (0.42)		183 (0.50)	
Type unknown		97 (0.22)		17 (0.24)		80 (0.22)	
Prior non-KI transplant	0	811 (1.8)	0	145 (2.0)	0	666 (1.8)	0.23^c^
Dialysis*	80	28,359 (64.5)	12	4,619 (64.3)	68	23,740 (64.5)	0.78^c^
Dialysis duration at transplant (months) *	166		26		140		0.054^c^
Preemptive		15,641 (35.6)		2,564 (35.8)		13,077 (35.6)	
>0 - 11.9		10,927 (24.9)		1,846 (25.7)		9,081 (24.7)	
12 - 23.9		7,890 (18.0)		1,284 (17.9)		6,606 (18.0)	
24 - 47.9		6,376 (14.5)		1,030 (14.4)		5,346 (14.5)	
48 - 71.9		2,085 (4.7)		298 (4.2)		1,787 (4.9)	
72+		995 (2.3)		147 (2.1)		848 (2.3)	
Peak cPRA*	538	10.3 ± 22.7	124	12.2 ± 25.7	414	9.9 ± 22.1	***<0.001^a2^***
Peak cPRA*	538		124		414		***<0.001^c^***
0		31,342 (72.0)		4,992 (70.6)		26,350 (72.2)	
1 - 19		4,580 (10.5)		714 (10.1)		3,866 (10.6)	
20 - 79		6,149 (14.1)		992 (14.0)		5,157 (14.1)	
80 - 97		1,138 (2.6)		252 (3.6)		886 (2.4)	
97 - 100		333 (0.76)		121 (1.7)		212 (0.58)	
Transplant year	0		0		0		***<0.001^c^***
2013		4,704 (10.7)		866 (12.0)		3,838 (10.4)	
2014		4,609 (10.5)		821 (11.4)		3,788 (10.3)	
2015		4,756 (10.8)		844 (11.7)		3,912 (10.6)	
2016		4,724 (10.7)		750 (10.4)		3,974 (10.8)	
2017		4,931 (11.2)		804 (11.2)		4,127 (11.2)	
2018		5,440 (12.3)		861 (12.0)		4,579 (12.4)	
2019		5,870 (13.3)		875 (12.2)		4,995 (13.5)	
2020		4,421 (10.0)		676 (9.4)		3,745 (10.2)	
2021		4,625 (10.5)		698 (9.7)		3,927 (10.6)	
Time on wait list days (months)	0		0		0		***<0.001^c^***
0		1,054 (2.4)		240 (3.3)		814 (2.2)	
>0 - 5.9		17,055 (38.7)		3,018 (41.9)		14,037 (38.1)	
6 - 11.9		10,252 (23.3)		1,677 (23.3)		8,575 (23.2)	
12 - 23.9		8,711 (19.8)		1,320 (18.3)		7,391 (20.0)	
24 - 47.9		5,409 (12.3)		731 (10.2)		4,678 (12.7)	
48+		1,599 (3.6)		209 (2.9)		1,390 (3.8)	
Transplant procedure type	0		0		0		0.86^c^
Left kidney		39,150 (88.8)		6,386 (88.8)		32,764 (88.8)	
Right kidney		4,930 (11.2)		809 (11.2)		4,121 (11.2)	

*Data not available for all subjects. Missing values: Education = 906; Primary insurance = 20; BMI = 179; Disease etiology = 12; Diabetes type = 10.

Dialysis = 80; Dialysis duration at transplant (months) = 166; Peak cPRA = 538.

Statistics presented as mean ± SD, N (column %).

p-values: ^a1^t-test, ^a2^Satterthwaite t-test, ^c^Pearson’s chi-square test.

BMI, body mass index; cPRA, calculated panel reactive antigen.Bold and italicized values suggest statistical significance , p <0.05.

Private insurance was slightly more common in the 0 HLA-DR mismatch group (56.4%) compared to the 1-2 HLA-DR mismatch group (55.5%) (p < 0.001). The 0 HLA-DR mismatch group had more recipients with glomerulonephritis (31.0%) and fewer with polycystic kidney disease (10.4%) compared to the 1-2 HLA-DR mismatch group (28.4% and 14.0%, respectively) (p < 0.001). There were more recipients without diabetes in the 0 HLA-DR mismatch group (71.5%) compared to the 1-2 HLA-DR mismatch group (69.5%) (p < 0.001).

The peak cPRA was higher in the 0 HLA-DR mismatch group compared to the 1-2 HLA-DR mismatch group (mean: 12.2 vs 9.9; p < 0.001). There was no significant difference in the preemptive transplant between the two groups, with 36% of subjects having received preemptive kidney transplants.

Donor age showed a slight variation, with a mean age of 43.1 ± 12.5 years in the 0 mismatch group compared to 43.8 ± 12.4 years in the 1-2 mismatch group. (p < 0.001) (**Table 2**). The proportion of female donors was lower in the 0 mismatch group (61.6%) compared to the 1-2 mismatch group (64.2%). Regarding race/ethnicity, a smaller percentage of Non-Hispanic white donors (64.6%) were observed in the 0 mismatch group compared to the 1-2 mismatch group (70.4%). Hypertension was noted in 4.5% versus 4.9% of donors, and the donor BMI was consistent at 27 kg/m^2^ in both groups. The relationship between the donor and recipient revealed notable differences: 75.7% of donors with no mismatch were related to the recipient, significantly higher than the 36.7% in the 1-2 mismatch group (p < 0.001). Compatibility in donor/recipient sex was also more common in the 0 mismatch group (48.8% versus 44.7%).

**Table 2:** Characteristics of kidney living donors by HLA-DR mismatches.

Factor	Overall (N=44,080)		0 HLA-DR mismatches (N=7,195)		1-2 HLA-DR mismatches (N=36,885)		p-value
	N missing	Statistics	N missing	Statistics	N missing	Statistics	
Donor age (years)	0	43.7 ± 12.4	0	43.1 ± 12.5	0	43.8 ± 12.4	***<0.001^a1^***
Donor age (years)	0		0		0		***0.007^c^***
18 - 39		17,194 (39.0)		2,900 (40.3)		14,294 (38.8)	
40 - 49		11,730 (26.6)		1,873 (26.0)		9,857 (26.7)	
50 - 59		10,144 (23.0)		1,673 (23.3)		8,471 (23.0)	
60+		5,012 (11.4)		749 (10.4)		4,263 (11.6)	
Donor sex	0		0		0		***<0.001^c^***
Female		28,128 (63.8)		4,433 (61.6)		23,695 (64.2)	
Male		15,952 (36.2)		2,762 (38.4)		13,190 (35.8)	
Donor race/ethnicity	0		0		0		***<0.001^c^***
Non-Hispanic white		30,609 (69.4)		4,645 (64.6)		25,964 (70.4)	
Hispanic white		6,562 (14.9)		1,322 (18.4)		5,240 (14.2)	
Black		4,157 (9.4)		641 (8.9)		3,516 (9.5)	
Other		2,752 (6.2)		587 (8.2)		2,165 (5.9)	
Donor BMI (kg/m2)	114	26.9 ± 4.1	11	27.0 ± 4.0	103	26.9 ± 4.1	0.12^a1^
Donor BMI (kg/m2)	114		11		103		0.41^c^
<20		1,443 (3.3)		223 (3.1)		1,220 (3.3)	
20 - 24.9		13,536 (30.8)		2,165 (30.1)		11,371 (30.9)	
25 - 29.9		18,812 (42.8)		3,120 (43.4)		15,692 (42.7)	
30 - 34.9		9,136 (20.8)		1,516 (21.1)		7,620 (20.7)	
35+		1,039 (2.4)		160 (2.2)		879 (2.4)	
Donor history of cigarette use	0	10,811 (24.5)	0	1,773 (24.6)	0	9,038 (24.5)	0.80^c^
Donor diabetes	51	11 (0.02)	12	1 (0.01)	39	10 (0.03)	0.99^d^
Donor hypertension	36	2,116 (4.8)	6	322 (4.5)	30	1,794 (4.9)	0.16^c^
Donor eGFR, 2021 (mL/min/1.73m^2^)	0	100.0 ± 15.6	0	101.0 ± 15.8	0	99.9 ± 15.5	***<0.001^a1^***
Donor eGFR, 2021 (mL/min/1.73m^2^)	0		0		0		***<0.001^c^***
120+		4,949 (11.2)		918 (12.8)		4,031 (10.9)	
105 - 119		12,924 (29.3)		2,185 (30.4)		10,739 (29.1)	
90 - 104		14,431 (32.7)		2,246 (31.2)		12,185 (33.0)	
75 - 89		9,384 (21.3)		1,483 (20.6)		7,901 (21.4)	
60 - 74		2,392 (5.4)		363 (5.0)		2,029 (5.5)	
Donor is related to the recipient	0	18,977 (43.1)	0	5,449 (75.7)	0	13,528 (36.7)	***<0.001^c^***
Donor/recipient are the same sex	0	20,017 (45.4)	0	3,511 (48.8)	0	16,506 (44.7)	***<0.001^c^***
Donor/recipient are both male	0	9,876 (22.4)	0	1,712 (23.8)	0	8,164 (22.1)	***0.002^c^***
Donor/recipient ABO incompatible	0	611 (1.4)	0	99 (1.4)	0	512 (1.4)	0.94^c^
Donor/recipient weight ratio	66		6		60		***<0.001^c^***
<0.8 (>20% undersized)		9,797 (22.3)		1,386 (19.3)		8,411 (22.8)	
0.8 - 1.2 (within 20% of recipient’s weight)		27,195 (61.8)		4,548 (63.3)		22,647 (61.5)	
>1.2 (>20% oversized)		7,022 (16.0)		1,255 (17.5)		5,767 (15.7)	
LDKPI (w/2021 eGFR)	239		31		208		***<0.001^c^***
<21		27,426 (62.6)		5,833 (81.4)		21,593 (58.9)	
21 - 40		10,826 (24.7)		993 (13.9)		9,833 (26.8)	
41 - 60		4,287 (9.8)		274 (3.8)		4,013 (10.9)	
61 - 80		1,118 (2.6)		58 (0.81)		1,060 (2.9)	
100+		184 (0.42)		6 (0.08)		178 (0.49)	

Statistics presented as mean ± SD, N (column %).

p-values: ^a1^t-test, ^a2^Satterthwaite t-test, ^c^Pearson’s chi-square test, ^d^Fisher’s Exact test.

BMI, body mass index; LDKPI, living donor kidney profile index; eGFR, estimated glomerular filtration rate.Bold and italicized values suggest statistical significance , p <0.05.

Donor eGFR (based on the race-free 2021 equation) was 101.0 ± 15.8 mL/min/1.73m^2^ in the 0 HLA-DR mismatch group versus 99.9 ± 15.5 mL/min/1.73m^2^ in the 1-2 HLA-DR mismatch group (p < 0.001). The LDKPI was lower in the 0 HLA-DR mismatch group, with 81% of the 0 HLA-DR mismatch group having an LDKPI score under 21 compared to 59% in the 1-2 HLA-DR mismatch group (p<0.001).

Conversely, no significant differences were observed in terms of donor history of cigarette use or prevalence of diabetes between the two groups. Donor/recipient weight ratio and the proportion of ABO incompatibility between donor and recipient showed no significant variation.

### Graft loss

**Figure 3:**
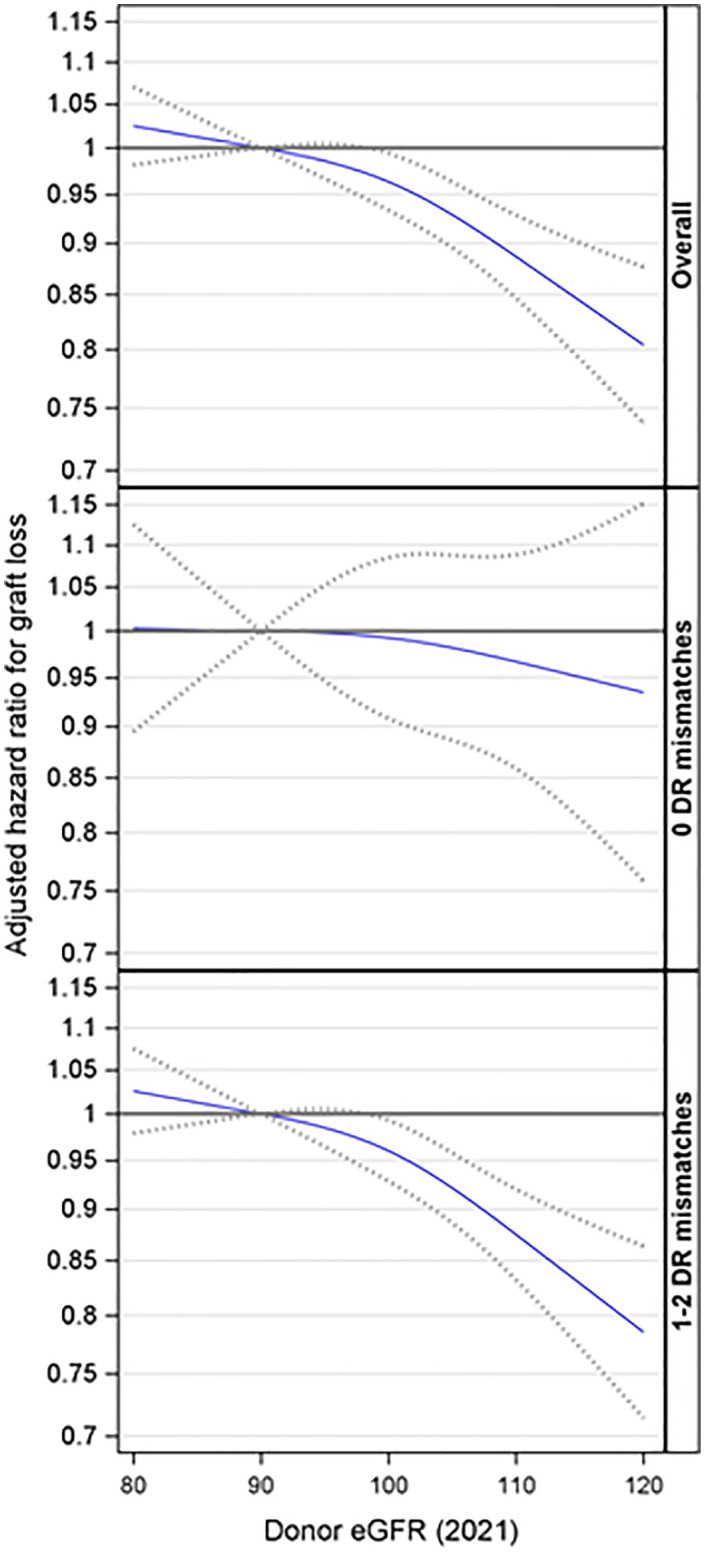
Association between donor eGFR and kidney graft loss, overall and by HLA-DR mismatch. The models were adjusted for recipient age, race/ethnicity, BMI, education, insurance, primary diagnosis, peak cPRA, time on dialysis, donor/recipient weight ratio, donor and recipient both being male, donor and recipient being of the same race, ABOi, and donor and recipient being related. eGFR, estimated glomerular filtration rate.

Based on the overall adjusted analysis, higher donor eGFR levels were associated with a decreased risk of graft loss (p < 0.001), with an adjusted hazard ratio (aHR) at eGFR=99 and 120 mL/min/1.73m^2^ of 0.97 (95% CI: 0.94, 0.99) and 0.80 (95% CI: 0.74, 0.88) compared to an eGFR=90 mL/min/1.73m^2^ (**Figure 3**). The interaction effect between donor eGFR and HLA-DR mismatch on graft loss was not statistically significant (p =0.67). However, the stratified models revealed that higher donor eGFR levels were associated with a decreased risk of graft loss in the presence of HLA-DR mismatch (p < 0.001), with an aHR at eGFR=99 and 120 mL/min/1.73m^2^ of 0.97 (0.94, 0.99) and 0.78 (0.71, 0.86) compared to an eGFR=90 mL/min/1.73m^2^ (**Figure 3**). However, there was no evidence of a significant association when there was no HLA-DR mismatch (p = 0.81).

**Figure 4:**
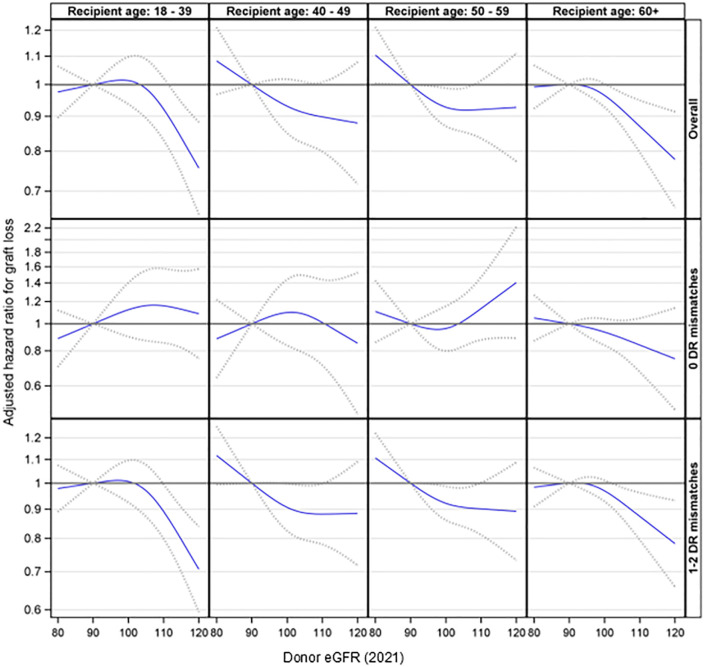
Association between donor eGFR and kidney graft loss by recipient age group, overall and by HLA-DR mismatch. The models were adjusted for recipient age, race/ethnicity, BMI, education, insurance, primary diagnosis, peak cPRA, time on dialysis, donor/recipient weight ratio, donor and recipient both being male, donor and recipient being of the same race, ABOi, and donor and recipient being related. eGFR, estimated glomerular filtration rate.

Further analyses were conducted to explore the interaction effect between donor eGFR and recipient age on graft loss. This interaction was not statistically significant (p= 0.24). Additionally, the three-way interaction involving donor eGFR, recipient age, and HLA-DR matches was not statistically significant (p= 0.87). However, the stratified analyses suggested that higher donor eGFR levels were associated with a reduced risk of graft loss among recipients aged 18-39 (p < 0.001) and 60 or older (p = 0.021) in the presence of an HLA-DR mismatch but not otherwise (**Figure 4**).

The results were consistent when using the 2009 race-included eGFR equation (**Supplementary Figures 1**, **2**).

### Patient death

**Figure 5:**
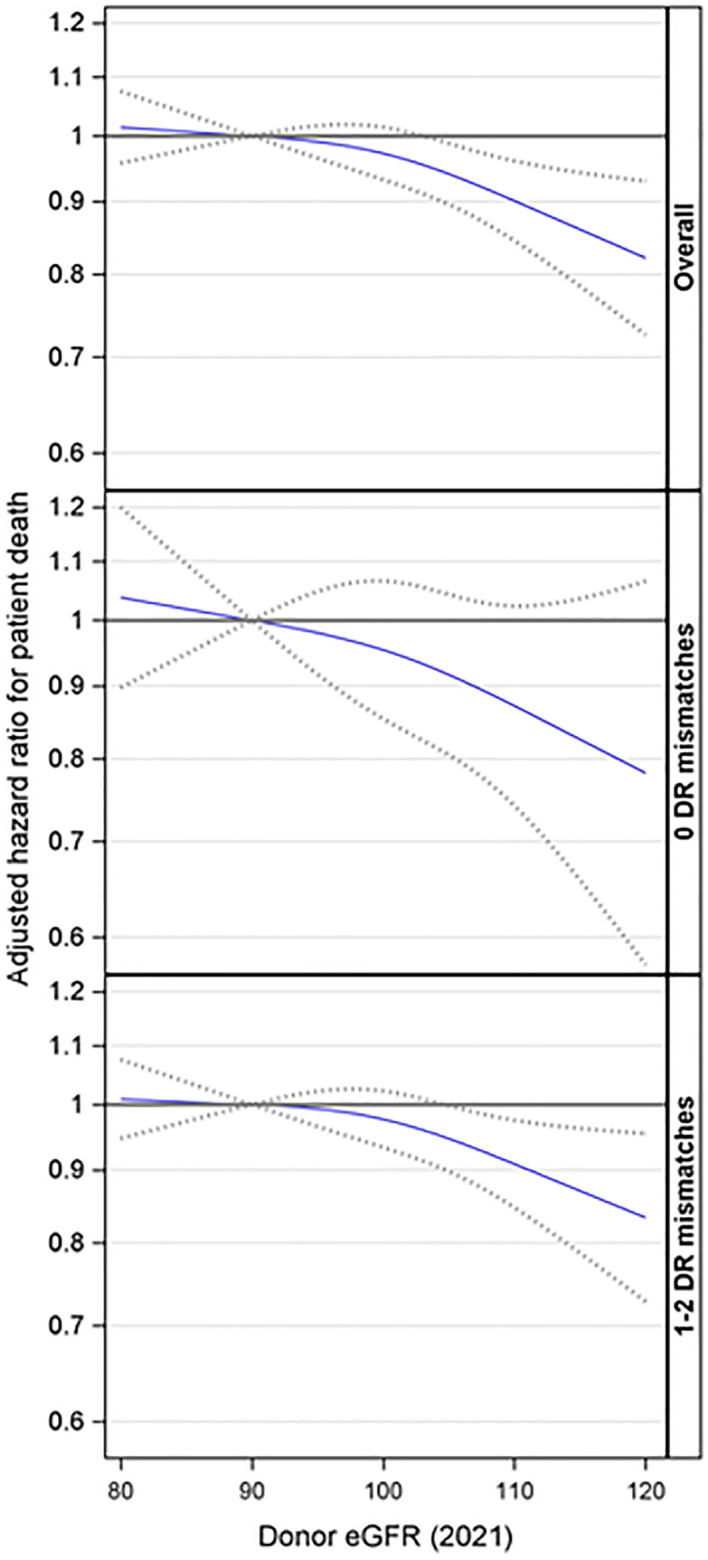
Association between donor eGFR and patient death, overall and by HLA-DR mismatch. The models were adjusted for recipient age, race/ethnicity, BMI, education, insurance, primary diagnosis, peak cPRA, time on dialysis, donor/recipient weight ratio, donor and recipient both being male, donor and recipient being of the same race, ABOi, and donor and being recipient related. eGFR, estimated glomerular filtration rate.

Based on the overall adjusted analysis, higher donor eGFR levels were associated with a decreased risk of patient death (p = 0.005), with an aHR at eGFR=104 and 120 mL/min/1.73m^2^ of 0.95 (95% CI: 0.90, 0.99) and 0.82 (95% CI: 0.73, 0.93) compared to an eGFR=90 mL/min/1.73m^2^ (**Figure 5**). The interaction effect between donor eGFR and HLA-DR mismatch on patient death was not statistically significant (p =0.52). However, the stratified models revealed that higher donor eGFR levels were associated with a decreased risk of patient death in the presence of an HLA-DR mismatch (p = 0.024), with an aHR at eGFR=105 and 120 mL/min/1.73m^2^ of 0.95 (0.89, 0.99) and 0.83 (0.73, 0.95) compared to an eGFR=90 mL/min/1.73m^2^ (**Figure 5**). However, there was no evidence of a significant association when there was no DR mismatch (p = 0.24).

**Figure 6:**
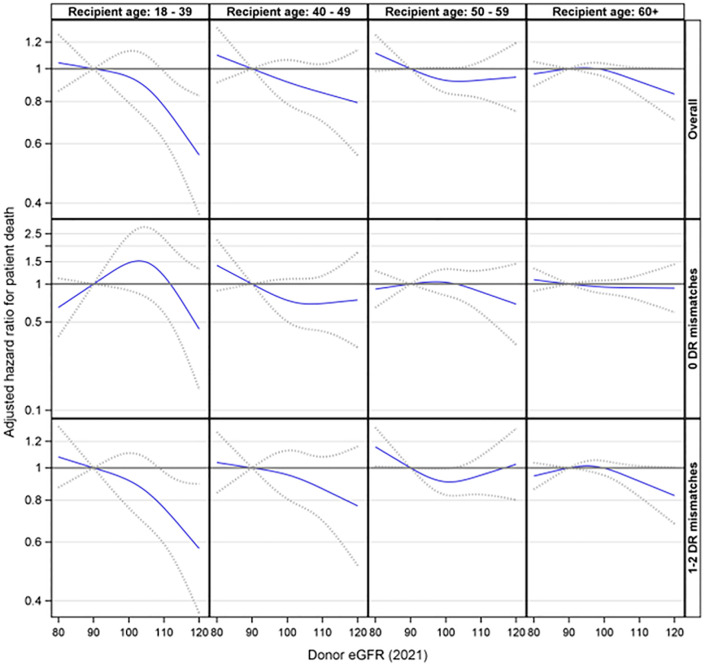
Association between donor eGFR and patient death by recipient age group, overall and by HLA-DR mismatch. The models were adjusted for recipient age, race/ethnicity, BMI, education, insurance, primary diagnosis, peak cPRA, time on dialysis, donor/recipient weight ratio, donor and recipient both being male, donor and recipient being of the same race, ABOi, and donor and recipient being related. eGFR, estimated glomerular filtration rate.

Further analyses were conducted to explore the interaction effect between donor eGFR and recipient age on patient death. This interaction was not statistically significant (p= 0.46). Additionally, the three-way interaction involving donor eGFR, recipient age, and HLA-DR matches was not statistically significant (p= 0.56). However, the stratified analyses suggested that higher donor eGFR levels were associated with a reduced risk of patient death among recipients aged 18-39 (p = 0.042) in the presence of an HLA-DR mismatch but not otherwise (**Figure 6**).

The results were consistent when using the 2009 race-included eGFR equation (**Supplementary Figures 3**, **4**).

## Discussion

This study highlights the complex interplay between donor eGFR and HLA-DR mismatches in the survival outcomes of grafts in LDKT. In the overall models, higher donor eGFR was associated with a lower risk of graft loss and patient death. These effects were evident for the DR mismatch group, but donor eGFR was not associated with survival in the 0 HLA-DR mismatch group. This highlights the importance of considering class 2 HLA-DR matching in conjunction with donor kidney function when matching living donors with recipients to optimize graft survival outcomes. 

In the current study, the recipient characteristics were similar in age, BMI, race, and preemptive transplant, with mild differences in sex and ethnicity. The donor age was also similar, averaging approximately 42 years, and eGFR values were comparable at approximately 100 ml/min. No significant differences were observed in terms of donor BMI, donor/recipient weight ratios, or the proportion of ABO incompatibility between donor and recipient.

The findings of our study add depth to the existing literature, as some previous studies, such as Fuggle et al. (2010) (7) and Casey (2015) (8), noted lesser or no significant impact of HLA mismatches on long-term graft survival in LDKTs but there was an effect due to living donation and higher GFR. Our study suggests that higher donor eGFR levels are associated with a reduced risk of graft loss in cases with HLA-DR mismatches, a finding not observed in cases without HLA-DR mismatches. This suggests the protective effects of immunological compatibility.

Furthermore, no HLA-DR mismatch with a related donor may be more closely aligned due to familial HLA similarities. Since the SRTR reports HLA typing at the antigen level rather than the molecular level, there may be more significant molecular-level differences in recipients with HLA-DR mismatches than those without.

The interaction effects involving donor eGFR, recipient age, and HLA-DR matches on graft loss did not reach significance. However, a stratified analysis suggested that higher donor eGFR levels were associated with a reduced risk of graft loss in the presence of an HLA-DR mismatch. This was especially significant for those aged 18-39, where HLA-DR matching played a larger role in prolonging graft survival. Thus, given a choice, HLA-DR matching is particularly important for younger recipients not only for prolonging graft survival but also for avoiding sensitization. Younger patients are more likely to need more than one kidney transplant during their lifetime and have a higher chance of becoming sensitized by a first failed transplant (13). This association was also observed in older recipients; it is possible that, in older patients, the reduced immune response and increased prevalence of competing risks such as cardiovascular disease or mortality may minimize the observed impact of an HLA-DR mismatch on graft outcomes. Conversely, in younger recipients, the stronger immune response may amplify the impact of an HLA-DR mismatch. Therefore, HLA-DR matching should be an important criterion for selecting their initial kidney transplant.

The operationalization of these insights could be particularly useful in settings with multiple donors and pre-selection for paired kidney exchanges. For example, in situations with non-directed donors where two recipients have an equal wait time, it could help choose which recipient would benefit most from the kidney based on HLA-DR matching. Furthermore, HLA-DR matching should be a critical criterion for selecting the initial kidney transplant for younger recipients, where the impact can be significant. For older patients waiting for a transplant, if the option of HLA-DR matching is not available, then the patient with the higher GFR could be considered. While HLA matching is important, the impact of waiting for an HLA-DR match needs to be weighed against the potential benefits of a timely transplant.

While our study leverages a large national database, it is not without limitations, chiefly its retrospective design and reliance on data from the SRTR, which are restricted to the traditional six major histocompatibility antigens. The OPTN is considering policies for adding additional non-traditional HLAs, which could further refine our understanding of antigen compatibility in transplant outcomes. We used estimated GFR equations, which may be less accurate than measured GFR.

Future studies should aim to include prospective data collection to validate our findings. Moreover, the influence of socioeconomic factors, recipient medication adherence, and other non-HLA antigens were not addressed in this study and could be significant. We advocate for cautious extrapolation and decision-making in each context.

## Conclusion

Our study suggests that higher donor eGFR levels are associated with a reduced risk of graft loss in cases with HLA-DR mismatches, a finding not observed in cases without HLA-DR mismatches. This suggests the protective effects of immunological compatibility. This was especially significant for those aged 18-39, where HLA-DR matching played a larger role in prolonging graft survival. Given a choice, HLA-DR matching is particularly important for younger recipients for not only prolonging graft survival but also avoiding sensitization.

## Data Availability

The original contributions presented in the study are included in the article/**Supplementary Material**. Further inquiries can be directed to the corresponding author.
