# Supplementary material for: Evaluating the impact of donor eGFR and HLA-DR mismatch on graft survival in living donor kidney transplants
Source: Front Nephrol. 2025 Jan 7;4:1518791. doi: 10.3389/fneph.2024.1518791 (PMC11747204; doi:10.3389/fneph.2024.1518791)
Supplement: Supplementary file 1 [file DataSheet1.docx]

**Supplemental Materials**

Table of Contents

[**Supplemental Figure 1. Association between 2009 CKD-EPI race-included donor eGFR and kidney graft loss, overall and by DR mismatch** 2](#_Toc170900343)

[**Supplemental Figure 2. Association between 2009 CKD-EPI race-included donor eGFR and kidney graft loss by recipient age group, overall and by DR mismatch** 3](#_Toc170900344)

[**Supplemental Figure 3. Association between donor 2009 CKD-EPI race-included eGFR and patient death, overall and by DR mismatch** 4](#_Toc170900345)

[**Supplemental Figure 4. Association between donor 2009 CKD-EPI race-included eGFR and patient death by recipient age group, overall and by DR mismatch** 5](#_Toc170900346)

# **Supplemental Figure 1. Association between 2009 CKD-EPI race-included donor eGFR and kidney graft loss, overall and by DR mismatch**


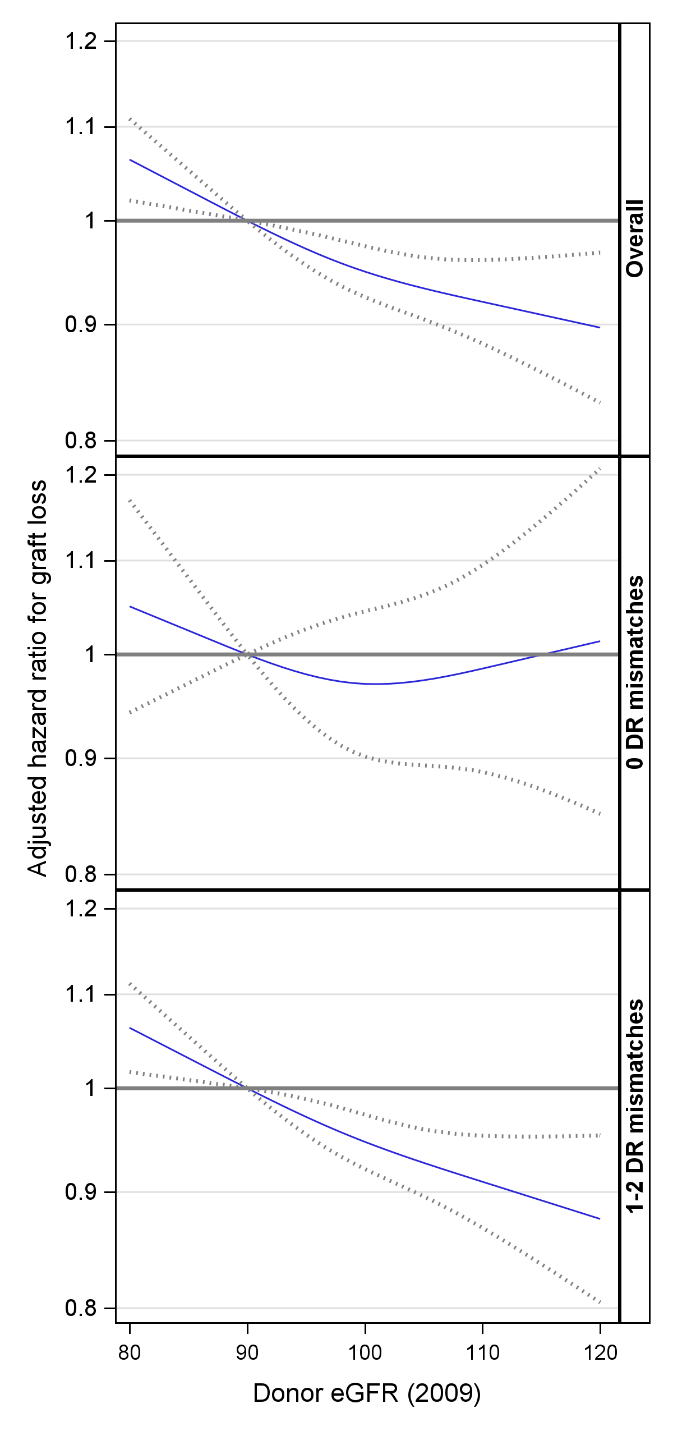


Models adjusted for recipient age, race/ethnicity, BMI, education, insurance, primary diagnosis, peak cPRA, time on dialysis, donor/recipient weight ratio, donor and recipient both male, donor and recipient of same race, ABOi, and donor and recipient related.

CKD-EPI: chronic kidney disease epidemiology collaboration; eGFR: estimated glomerular filtration rate

# **Supplemental Figure 2. Association between 2009 CKD-EPI race-included donor eGFR and kidney graft loss by recipient age group, overall and by DR mismatch**


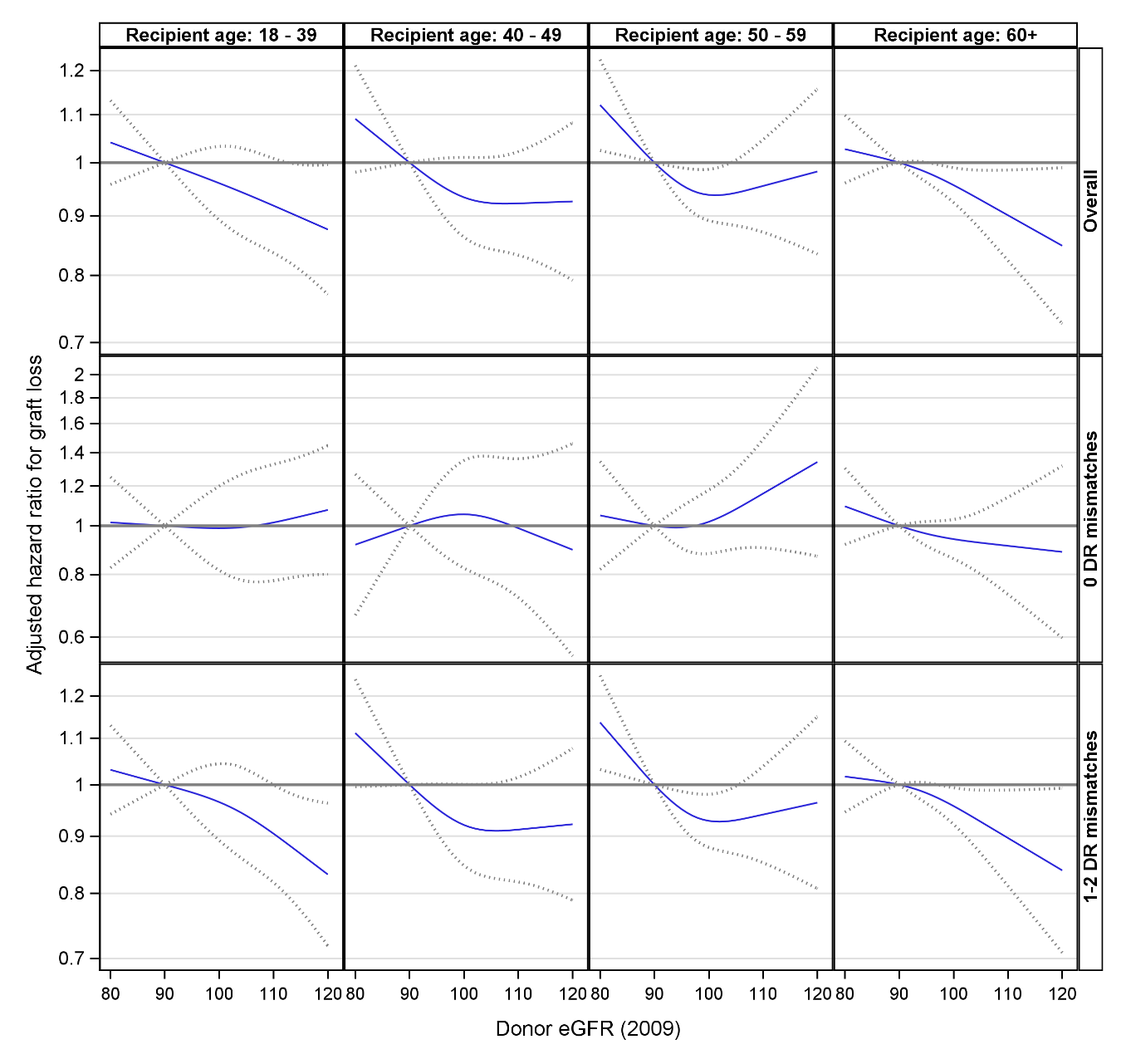


Models adjusted for recipient age, race/ethnicity, BMI, education, insurance, primary diagnosis, peak cPRA, time on dialysis, donor/recipient weight ratio, donor and recipient both male, donor and recipient of same race, ABOi, and donor and recipient related.

CKD-EPI: chronic kidney disease epidemiology collaboration; eGFR: estimated glomerular filtration rate

# **Supplemental Figure 3. Association between donor 2009 CKD-EPI race-included eGFR and patient death, overall and by DR mismatch**


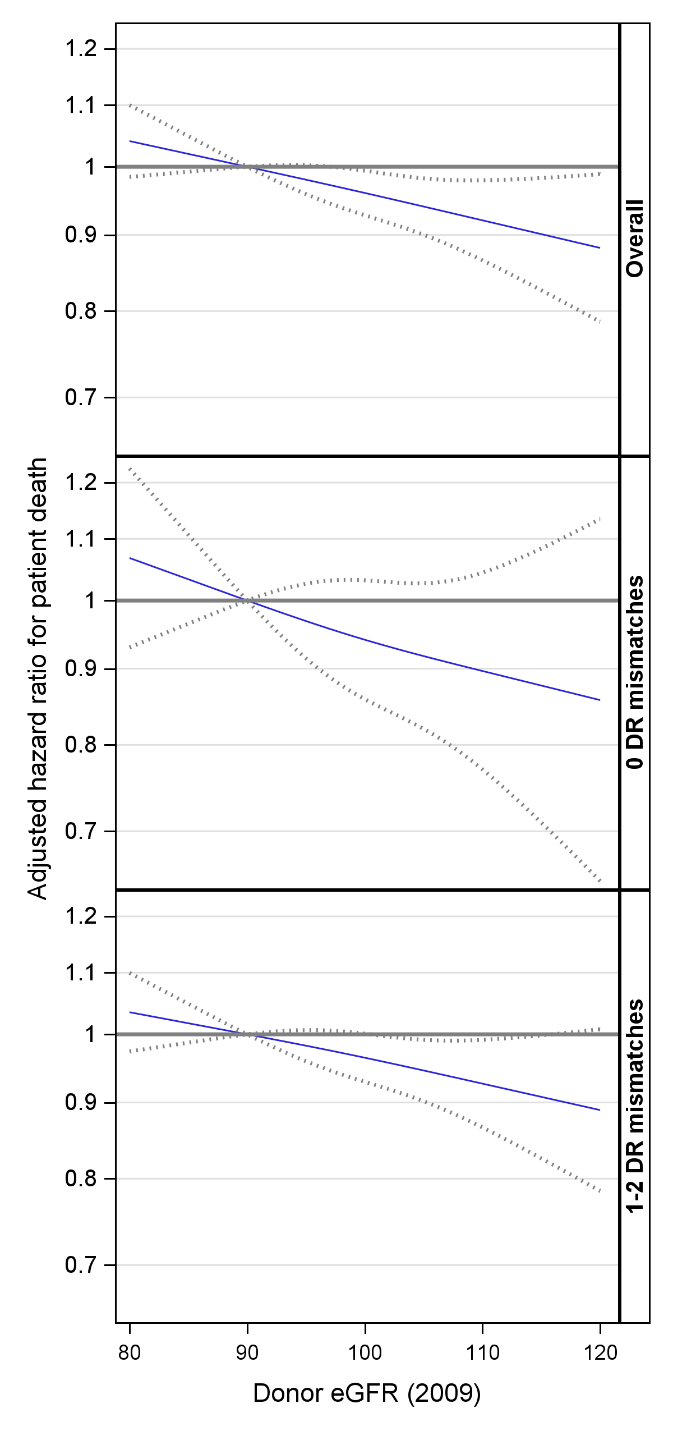


Models adjusted for recipient age, race/ethnicity, BMI, education, insurance, primary diagnosis, peak cPRA, time on dialysis, donor/recipient weight ratio, donor and recipient both male, donor and recipient of same race, ABOi, and donor and recipient related.

CKD-EPI: chronic kidney disease epidemiology collaboration; eGFR: estimated glomerular filtration rate

# **Supplemental Figure 4. Association between donor 2009 CKD-EPI race-included eGFR and patient death by recipient age group, overall and by DR mismatch**


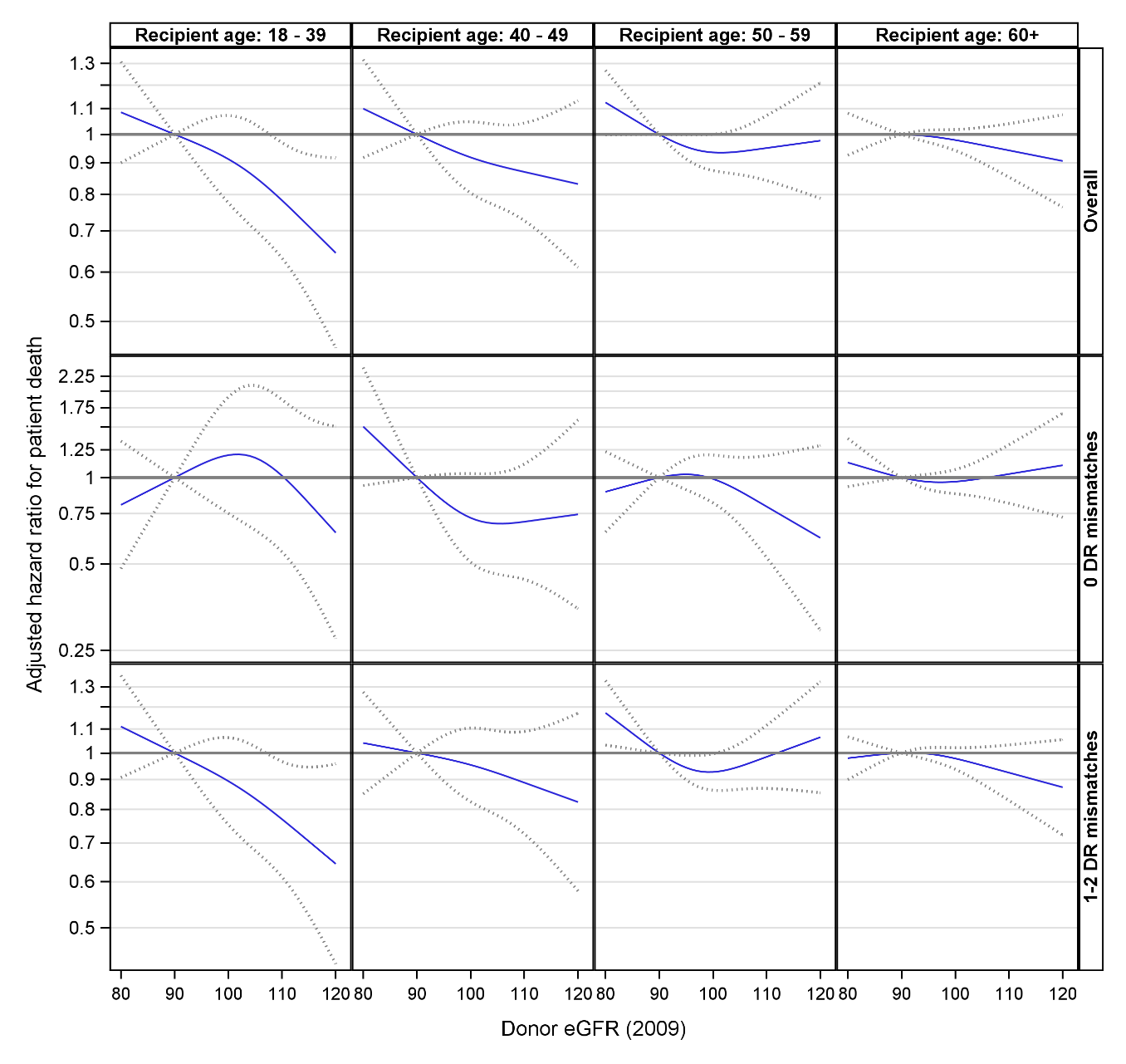


Models adjusted for recipient age, race/ethnicity, BMI, education, insurance, primary diagnosis, peak cPRA, time on dialysis, donor/recipient weight ratio, donor and recipient both male, donor and recipient of same race, ABOi, and donor and recipient related.

CKD-EPI: chronic kidney disease epidemiology collaboration; eGFR: estimated glomerular filtration rate
